# Community-level dietary intake of sodium, potassium, and sodium-to-potassium ratio as a global public health problem: a systematic review and meta-analysis

**DOI:** 10.12688/f1000research.122560.1

**Published:** 2022-08-18

**Authors:** Farapti Farapti, Putri Hersya Maulia, Chusnul Fadilla, Niwanda Yogiswara, Purwo Sri Rejeki, Muhammad Miftahussurur, Hazreen Abdul Majid

**Affiliations:** 1 Department of Nutrition, Faculty of Public Health, Universitas Airlangga, Surabaya, East Java, 60115, Indonesia; 2 Doctoral Program of Medical Science, Faculty of Medicine, Universitas Airlangga, Surabaya, Indonesia; 3 Faculty of Medicine, Universitas Airlangga, Surabaya, East Java, 60132, Indonesia; 4 Physiology Division, Department of Physiology and Biochemistry, Faculty of Medicine, Universitas Airlangga, Surabaya, East Java, 60132, Indonesia; 5 Institute of Tropical Disease, Universitas Airlangga, Surabaya, East Java, 60115, Indonesia; 6 Gastroentero-Hepatology Division, Department of Internal Medicine, Faculty of Medicine, Universitas Airlangga, Surabaya, East Javva, 60132, Indonesia; 7 Department of Social and Preventive Medicine, Faculty of Medicine, University of Malaya, Kuala Lumpur, Selangor, 50603, Malaysia

**Keywords:** sodium, potassium, sodium-to-potassium ratio, systematic review, hypertension

## Abstract

**Background:** Widespread adoption of a westernized diet represents a major lifestyle change characterized by substantially higher sodium consumption and lower potassium intake, which is related to cardiovascular morbidity.

**Methods:** We performed a systematic review and meta-analysis over published studies in accordance with quantifying the dietary intake of sodium and potassium of the universal population across the world. The PubMed, EMBASE, Cochrane Library, and Google Scholar databases were used to find research that pronounced 24-hour urinary sodium or potassium excretion (reference period: 2014–2021). The effect size was estimated using the fixed-effect model; sub-group analysis become accomplished to determine urinary sodium and potassium excretion disaggregated by geographical location. Publication bias became evaluated the usage of graphical funnel plot. Data analysis was performed using STATA 16.

**Results:** Forty-three studies (n= 62,940) qualified the selection criteria. The mean urinary excretion of sodium and potassium was 156.73 mmol/24h [95% confidence interval (CI), 148.98–164.47] and 48.89 mmol/24 h (95% CI, 43.61–54.17), respectively; the mean urinary sodium/potassium ratio was 3.68 (95% CI, 2.96–4.40).

**Conclusions:** This updated systematic review highlights excessively high dietary intake of sodium and low intake of potassium at the community level in most parts of the world. The urinary Na/K ratio exceeded the level recommended by the WHO guidelines.

## Introduction

Sodium (Na) and potassium (K) are the major electrolytes of the extracellular fluid and intracellular fluid, respectively. Both these nutrients circulate a crucial role in normal biological functioning such as regulation of body fluids, active transport of molecules across the cell membrane, maintenance of osmotic equilibrium, and acid-base balance. ^1^ Sodium and potassium are naturally occurring nutrients in a variety of foods. Processed food and condiments typically have a high sodium content, while fresh fruits, vegetables, and nuts are rich in potassium. ^2^^,^^3^ Industrialization and adoption of the westernized dietary patterns have contributed to the increased prevalence of obesity, metabolic diseases, and cardiovascular diseases. ^4^^,^^5^ The switch from a traditional diet to westernized dietary pattern represents a major lifestyle change leading to the substantial increase in sodium consumption and decline in potassium intake. ^4^ The Asian diet is already typically a high-salt diet, however westernization has further increased sodium levels, making the consumption of salt in Asia greater. ^6^

Several randomized clinical studies and systematic reviews have demonstrated the effect of sodium and potassium intake on non-communicable diseases. ^7^^,^^8^ Low potassium consumption and high sodium intake have been shown to be related to the extended danger of stroke, hypertension, and obesity. ^4^^,^^9^^,^^10^ The sodium-to-potassium (Na/K) ratio in diet showed a more potent affiliation with blood pressure (BP) than both sodium or potassium alone. ^11^^,^^12^ The World Health Organization (WHO) recommends a wholesome diet pattern to prevent the onset of diet-related diseases. Foods containing high potassium content and limited sodium content are particularly recommended. ^13^^,^^14^ Moreover, reducing sodium intake and increasing potassium consumption have been recognized as a concern intervention to lessen non-communicable illnesses. ^15^^,^^16^ In reality, most populations consume less than the recommended level of potassium and above the advocated level of sodium; the ratio of sodium to potassium typically ranges from 1 to 2. ^11^^,^^17^^,^^18^ According to the WHO, almost all countries report high sodium intake and potassium deficiency in the general population. ^15^^,^^16^

Several strategic interventions have been implemented to improve the intake of sodium and potassium. WHO has formulated recommendations to reduce population-wide dietary salt intake and has set a target of 30% reduction in salt intake across the globe by 2025. ^15^ WHO also recommends potassium intake of at least 90 mmol/day. ^16^ Moreover, the current guidelines recommended a Na/K ratio of approximately one. Despite these interventions, several studies have documented that most people still consume high-sodium and low-potassium foods in their diets. ^15^^,^^16^

A systematic review characterized the population-wide intake of these nutrients in China. ^19^ To our knowledge, no systematic review and meta-analysis has comprehensively assessed the sodium and potassium intake among populations across the world, as well as analyzed their relationship to other health problems at the community level, or their correlation to other community-level health issues. In this study, we attempt to systematically investigate the dietary consumption of sodium and potassium among adults at the community level across the world. We performed a systematic literature review and meta-analysis of data pertaining to 24-hour urinary excretion of sodium and potassium among healthy adult subjects. The objective was to quantify the dietary intake of sodium and potassium as a public health problem at the community level.

## Methods

### Database search strategy

We conducted a systematic review and meta-analysis of observational studies in a match to the Preferred Reporting Items for Systematic Review and Meta-Analysis (PRISMA) statement. ^93^ The literature search for this study was conducted from June to December 2021. The PubMed, EMBASE, Cochrane Library, and Google Scholar databases were used to search for applicable studies published between January 2014 and December 2021. The following search terms were used to retrieve articles: (Sodium Chloride OR Sodium OR salt OR Potassium OR sodium-potassium ratio OR sodium to potassium ratio) AND (dietary OR intake OR urinary). The detailed electronic search strategy is available in our review protocol. In addition, the reference lists of the retrieved articles were manually searched to obtain applicable articles. The protocol of this review has been published in the International Prospective Register of Systematic Reviews (CRD42022279435).

### Study selection and data extraction

Studies were considered eligible if they qualified the following criteria: (1) observational studies including cross-sectional and cohort studies published during the period 2014–2021; (2) study population: healthy subjects who underwent measurement of 24-hour urinary excretion of both sodium and potassium; (3) availability of full-text articles; and (4) language of publication: English. The exclusion criteria were: (1) case reports, review articles, commentaries, and letters; (2) outcomes not relevant to this study; or (3) subjects with comorbid diseases. The primary data were extracted from the articles into a spreadsheet using Microsoft Excel version 16. Data concerning to following variables were extracted: call of the primary author, year of publication, country of subjects, study design, sample size, patient characteristics and setting, the method used for determining urinary sodium, and potassium, urine sodium and potassium concentration **.** All reported concentrations of sodium and potassium were transformed into millimoles. The principal summary measures were the standard error mean (SEM) of sodium and potassium excretion. If SEM became not mentioned, we measured the same from the standard deviation and the number of samples. ^20^

### Quality assessment

Methodological quality of the included studies was assessed using assessment scale for non-randomized study developed by Newcastle-Ottawa (NOS). The assessment consisted of several aspects such as the selection, comparability, and outcome of the study. The database search, study selection, data extraction, and appraisal were independently performed by four authors (F.R., P.H.M., C.F., and N.Y.). Any differences of opinion were resolved by consensus and/or by consulting three senior investigators (H.A.M., M.M., and P.S.R.).

### Data analysis

In case of no significant heterogeneity among the included research (assessed by I ^2^ test), the fixed-effect model was used to calculate the estimated effect size for mean difference (MD). I ^2^ > 50% is taken to indicate the importance of significant heterogeneity. For significant heterogeneity, the random-effects model became used. Subsequently, subgroup analysis was conducted to specify sodium and potassium excretion disaggregated by age-group (children and adults) and geographical location (grouped by continent). Publication bias was measured using a graphical funnel plot. All analyses were done using STATA 16.0 (StataCorp LLC, College Station, Texas).

## Results

The database search produced a total of 6114 records. After elimination of duplicate records and manual search of the reference lists of the relevant articles, a total of 3450 abstracts were screened. Of these, 496 articles were subjected to full-text review, of which 357 were excluded due to various excuses. Finally, a total of 43 studies that reported 24-hour urinary sodium and potassium excretion were included in the study.

Supplementary Table 2 (see *Extended data* ^93^) summarizes the characteristics of the 43 studies and reports the 24-hour urinary sodium, potassium, and sodium-to-potassium (Na/K) ratio among adults based on continent. The studies included 18 from Asia, 9 from Europe, 8 from United States, 6 from Africa, and 2 from Australia. The majority of studies included both men and women; however, two studies used just a female population. ^11^^,^^21^ Most of the reports included in our meta-analysis used a combination of 24-hour urine collection and dietary assessment. ^11^^,^^21^^–^^33^ Likewise, the studies by Du *et al*. (2014) ^17^ and Morrisey *et al*. (2020) ^34^ used the spot urine method in combination with a dietary survey. Most of the included studies analyzed the correlation between the intake of sodium and potassium with blood pressure. ^11^^,^^17^^,^^25^^,^^34^^–^^42^ In addition, several studies discussed both blood pressure and/or obesity as outcomes. ^22^^,^^43^^–^^47^

**Figure 1.**
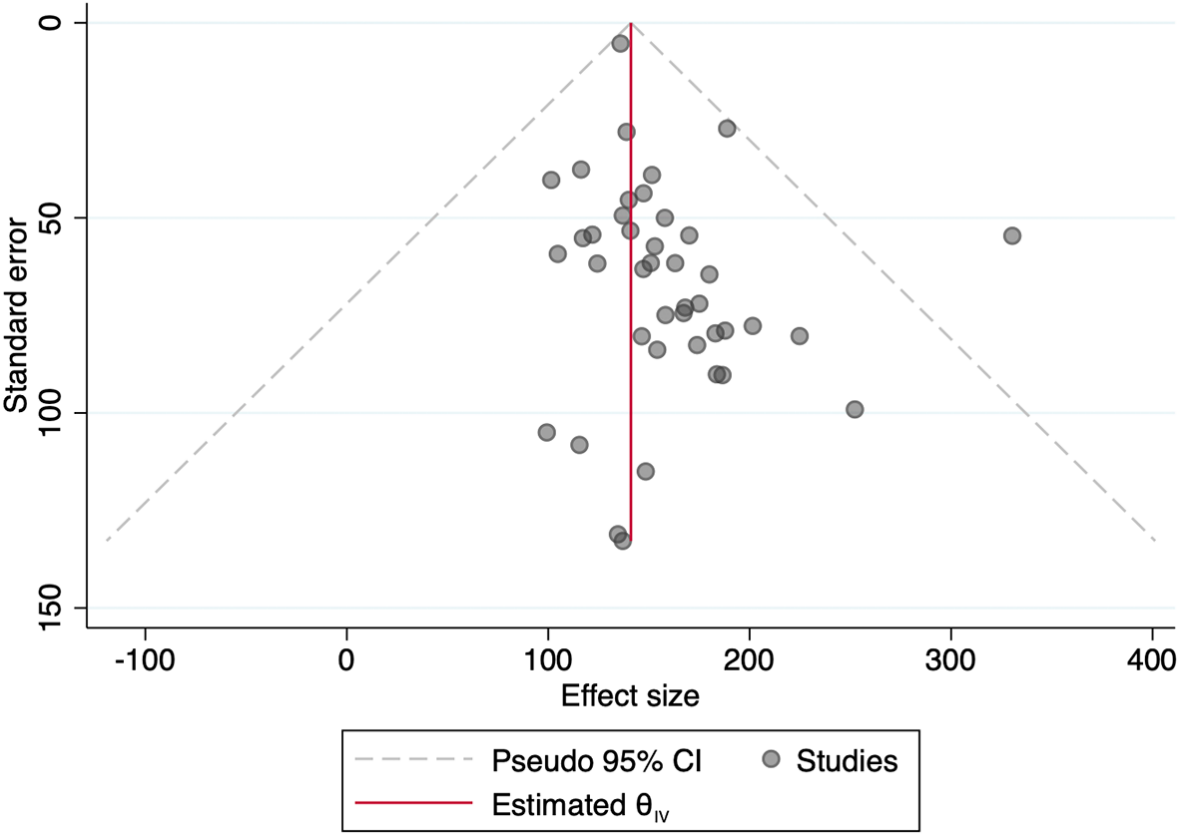
Funnel plot of the included studies using urinary sodium excretion outcome.

Figure 1 showed funnel plot of the included studies. Because of significant heterogeneity in a number of the included studies (I ^2^ 99.51%, p < 0.001), the random-effect sample was used for meta-analysis. No significant publication bias became discovered through the funnel plot based on the mean sodium excretion.

Table 1 summaries the meta-analysis and presents demonstrates that the majority of the studies were done in Asia (42%) followed by Europe and America (21% and 19%, respectively). Overall, the mean sodium excretion was 156.73 mmol/24 h [95% confidence interval (CI), 148.98–164.47] and the mean urinary potassium was 48.89 mmol/24 h (95% CI, 43.61–54.17). The highest mean sodium excretion was observed in Asia [175.72 mmol/24 h (95% CI, 1760.48–190.97), while the lowest mean sodium excretion was observed in Africa [137.9 mmol/24 h (95% CI, 119.42–156.56). The continent with the highest mean potassium excretion were Australia [73.11 mmol/24 h (95% CI 65.58–80.64)] followed by Europe [61.1 (95% CI, 51.59–70.61)]. The lowest potassium excretion was found in Africa [mean: 33.82 mmol/24 h (95% CI, 21.03–46.61)]. Urinary Na/K ratio was extracted from 27 (63%) out of 43 studies; the mean urinary Na/K ratio was 3.68 (95% CI, 2.96–4.40). The top urinary Na/K ratio was reported from Asia [mean: 5.03 (95% CI, 4.43–5.63)] while the lowest urinary Na/K ratio was reported from Australia [mean: 1.56 (95% CI, 1.09–2.03)].

**Table 1.** Summary of the meta-analysis.

Country	n	Sodium excretion (mmol/24 h)		Potassium excretion (mmol/24 h)		Sodium/potassium	
		ES	95% CI	ES	95% CI	ES	95% CI
All countries	43	156.73	(148.98-164.47)	48.89	(43.61-54.17)	3.68	(2.96-4.40)
Asia	18	175.72	(160.48-190.97)	39.92	(36.13-43.71)	5.03	(4.43-5.63)
Europe	9	152.96	(143.88-162.03)	61.1	(51.59-70.61)	2.62	(1.86–3.39)
America	8	139.94	(136.39-143.50)	56.25	(49.77–62.74)	2.2	(1.13–3.28)
Africa	6	137.99	(119.42-156.56)	33.82	(21.03-46.61)	3.52	(2.7–4.33)
Australia	2	141.03	(128.68–153.37)	73.11	(65.6–80.6)	1.56	(1.09–2.03)

ES = Effect size, 95% CI = 95% Confidence Interval.

**Figure 2.**
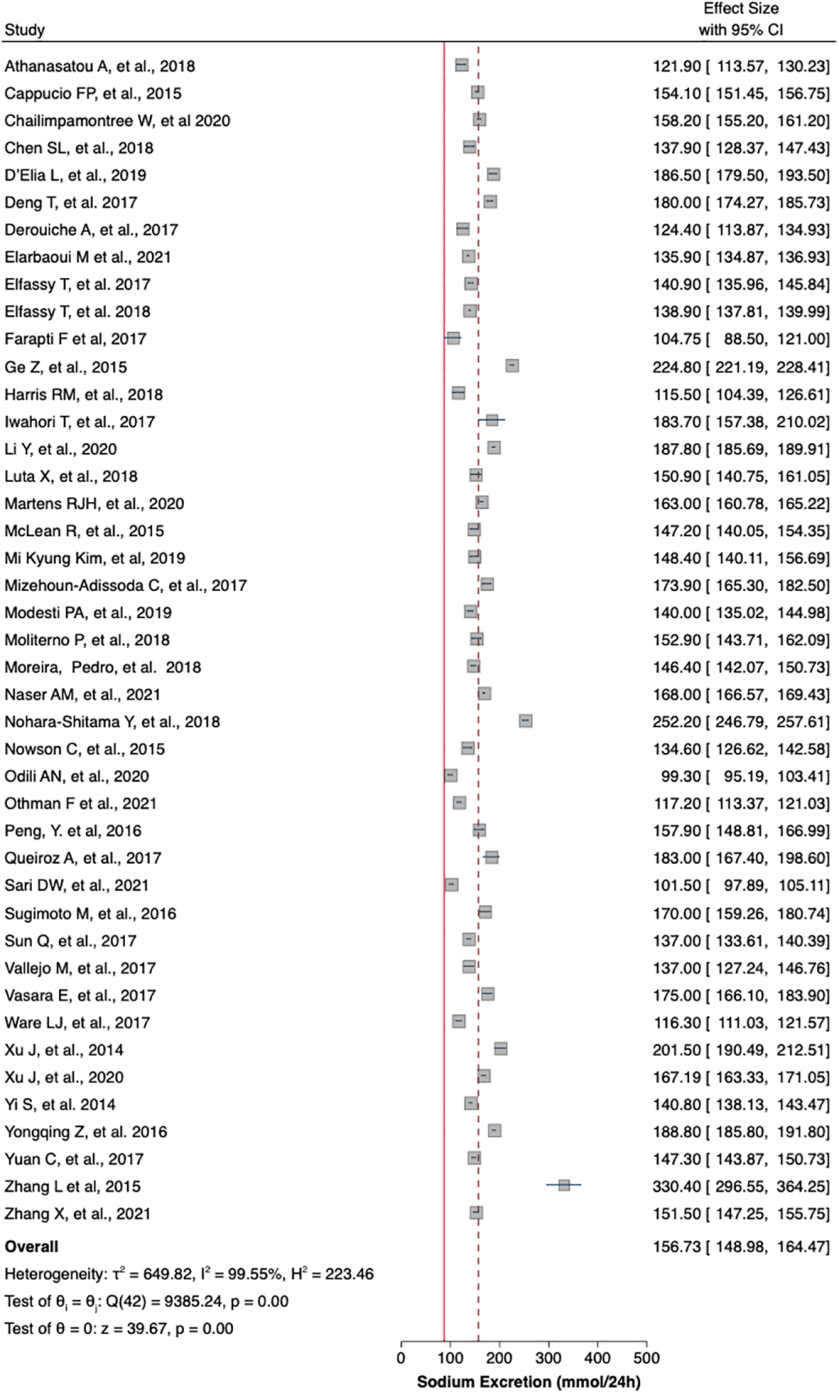
Mean urinary sodium excretion (mmol/24 h). The overall effect size estimate is represented by the red dashed line

**Figure 3.**
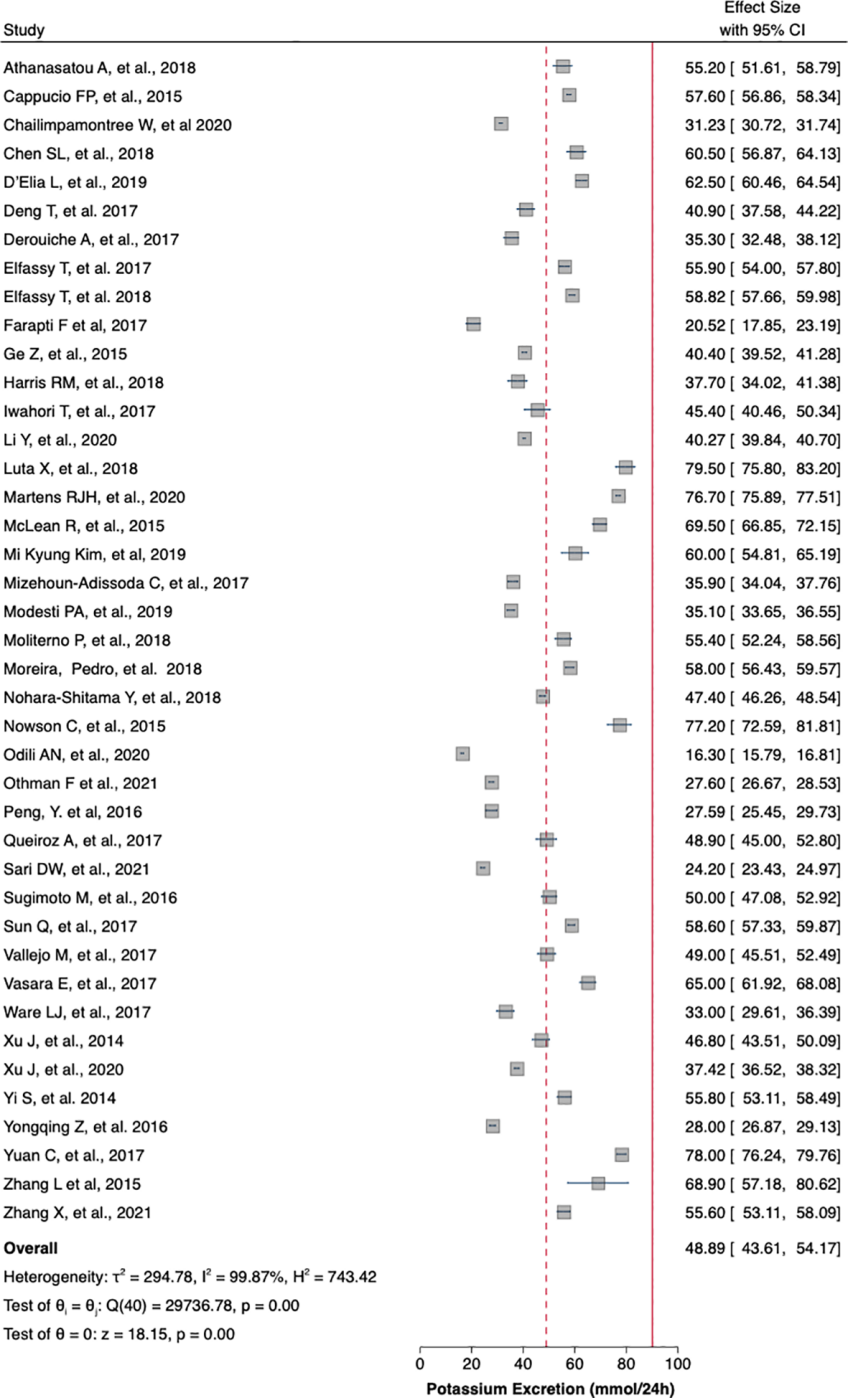
Mean urinary potassium excretion (mmol/24 h). The overall effect size estimate is represented by the red dashed line.

**Figure 4.**
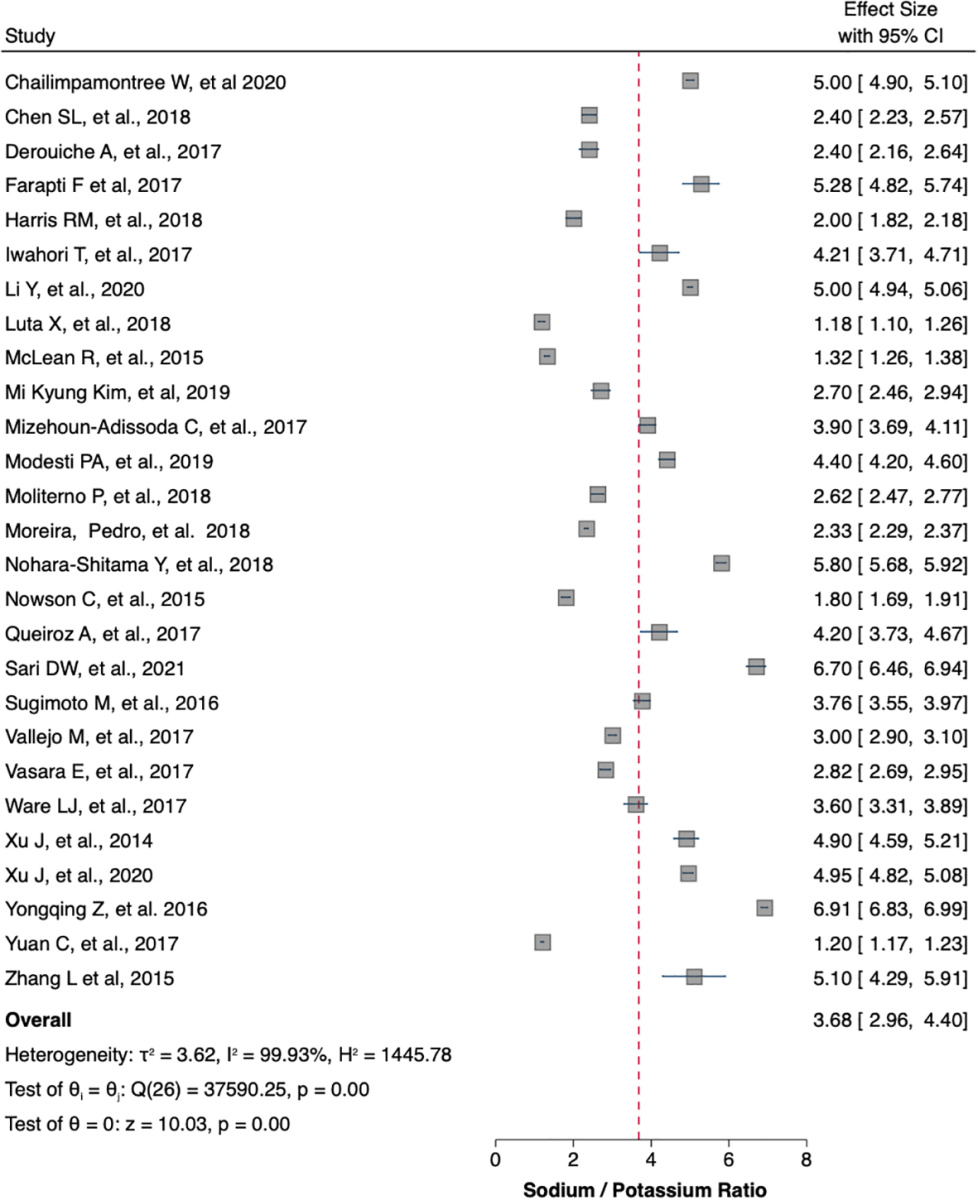
Sodium-potassium ratio (mmol/mmol). The overall effect size estimate is represented by the red dashed line.

The mean urinary sodium, urinary potassium, and sodium-potassium ratio were summarized as forest plots presented in Figure 2, Figure 3, and Figure 4 respectively. The overall effect size estimate is represented by the red dashed line. We observed heterogeneity among these studies and all forest plots showed I ^2^ > 50%, meaning significant heterogeneity. Figure 2, Figure 3, and Figure 4 demonstrated significant heterogeneity among the study that analyzed urinary sodium excretion, urinary potassium, and sodium-potassium ratio, respectively. Consequently, the random-effects model was used in these studies.

## Discussion

The more reliable methods for assessing dietary intake of sodium and potassium are typically difficult to perform, whereas the more convenient methods are less reliable. ^48^ The methods used to measure the intake of these nutrients including dietary survey, spot urine, and 24-hour urine collection. ^48^^–^^50^ The gold standard for assessment of Na and K intake is 24-hour urine collection; nevertheless, it is costly, cumbersome, and difficult to implement. ^48^ In our meta-analysis, we included all studies that used 24-hour urinary collection for assessment of Na and K intake. This is because it is crucial to acquire accurate measures at the populace level. It is important to note that while 24-hour urinary excretion is the best method, studies commonly employ a combination of methods including urine collection and dietary assessment. This approach is used to confirm the subjects’ dietary habits and to identify food sources of these nutrients. Moreover, the WHO recommends that 24-hour urine collection should be combined with a dietary evaluation. ^15^^,^^16^ Most of the reports included in our meta-analysis used a combination of 24-hour urine collection and dietary assessment. ^11^^,^^21^^–^^33^ Likewise, the studies by Du *et al*. (2014) ^17^ and Morrisey *et al*. (2020) ^34^ used the spot urine method in combination with the dietary survey.

Most of the research included in our meta-analysis was conducted in Asia, particularly in China and Japan. ^17^^,^^24^^–^^26^^,^^35^^,^^40^^,^^51^^–^^58^ In both these countries, there is a focus on these nutrients owing to the typically high-salt diet. Indeed, a systematic review and meta-analysis by Tan *et al.* (2019) ^19^ found a wide variety of research which had targeted the intake of these nutrients since the 1980s. Furthermore, long-term prospective cohort studies have assessed the intake of these nutrients among adult Chinese and Japanese population for 18 years and 24 years, respectively. ^17^^,^^59^ In our systematic review and meta-analysis, we only included observational studies that enrolled healthy subjects. Most of the included studies analyzed the correlation between the intake of sodium and potassium with blood pressure. ^11^^,^^17^^,^^25^^,^^34^^–^^42^ In addition, several studies discussed both blood pressure and/or obesity as outcomes, ^22^^,^^43^^–^^47^ while other studies calculated the mean sodium and potassium intake compared to the standard recommendation. ^21^^,^^23^^,^^24^^,^^28^^–^^32^^,^^58^^,^^60^^–^^66^ Not all studies included the urinary the Na/K ratio in their analysis. In most studies, Na/K ratio confirmed a more potent affiliation with incident hypertension than the intake of sodium or potassium by itself. ^11^^,^^17^^,^^58^

### Sodium intake

In this study, the mean sodium intake among adults was 156.73 (148.98–164.47) mmol/d, which is equivalent to the daily intake of 3.6 g sodium or 9.17 g salt; the highest intake was in the Asian population. This result is aligned with that of a preceding meta-analysis of research conducted in China wherein the mean sodium intake was found to be 189.07 mmol. ^19^ The China Health and Nutrition Survey cohort includes 16,869 adults aged 20–60 years which were followed from 1991 to 2009; the results showed that despite the decreasing trend observed over successive years, sodium intake was still double the level recommended by the Institute of Medicine, an American nonprofit, non-governmental organization. ^1^ The decline in sodium intake has been most marked in northern China since the 2000s. Despite the decrease, more than half of the working age population still has a habit of consuming a large amount of sodium in the past decade. ^17^ A systematic review by Bernstein *et al.* (2010) ^67^ examined the trends in urinary sodium in the United States during the period 1957–2003; the results showed that the average sodium excretion in 2003 was 3526 ± 75 mg. Sodium consumption in the US adult population looks to be well above the currently recommended levels and does not seems to have decreased with time. This phenomenon might be caused by processed foods and more salt added to foods such as poultry, meat and fish. ^14^^,^^68^ A recent study reported that the population-level sodium intake across the world is well greater than minimal physiological requirements. In addition, in many nations, the sodium intake is a great deal greater than each day intake recommended by the WHO. ^13^

WHO has recognized the third reduction in population salt consumption as one of the primary objectives in its global action plan for the prevention and management of non-communicable diseases. ^69^ Salt reduction has been shown to be the most effective intervention, and in a few cases, fee-saving interventions to reduce the developing burden of non-communicable diseases. Processed foods account for up to 95% of dietary salt intake in the United Kingdom compared to under three-quarters in Japan (20% from soy sauce alone). Otherwise, home-cooked foods account for up to 76% of dietary salt intake in south China. Webster *et al.* (2014) ^6^ provided a comprehensive overview of the progress made in working with the food industry to lessen the salt content in processed items. Currently, a total of 59 countries (80% of which implement national strategies for salt reduction) include programs to work with the food industry. Gupta *et al.* (2018) ^69^ assessed the perspectives over a range of stakeholders concerning improvement on an India-specific salt reduction strategy. Based on the barriers and facilitators, several of the recommendations were around consumer’s awareness, promoting salt reduction in the processed food industry while also implementing customer-friendly product labeling. According to Trieu *et al.* (2015), ^70^ up to 75 countries now have national salt reduction strategies, twice the number recorded at a similar review held in 2010. Most programs have multiple disciplines; twelve countries reported reduced salt consumption, 19 countries reduced dietary salt intake, and 6 reported improved nutrition knowledge, habits, or attitudes towards salt. Recent data showed that public health interventions to improve awareness also have a great impact in decreasing salt intake in the population. ^71^ However, sodium is a naturally occurring nutrient into a vast range over foods, in addition, processed foods account for a large percentage of dietary salt intake. Consequently, although individuals are trying to lessen their salt intake by not adding salt to food, they’re nonetheless likely to be eating already sodium-rich foods. This underlines the challenge in reducing salt consumption at the population level.

### Potassium intake

In our study, the mean potassium excretion in adults was 51.36 mmol/d, equivalent to 2 g/d. The highest mean potassium excretion was found in Australia while the lowest potassium excretion was found in Africa. In keeping with the latest international and national surveys, only a few populations comply with the recommended consumption of potassium. The Prospective Urban Rural Epidemiology (PURE) research assessed the potassium intake using a single morning spot urine test; the results showed that most of the people of Asian populations consume <2 g of potassium per day. ^68^ In the northern states of the US and Europe, the average potassium intake was 2.5–2.7 g/day, while in Africa and the southern states of the US, the average was ≤ 2 g/day. The estimate for Africa was recently validated in a study carried out in Benin, an African country with an excessive prevalence of high blood pressure in both rural and urban regions. ^16^ Data from the Swiss Salt Survey, showed the average potassium consumption in the general population was 2.6 g/day, based on 24-hour urine collection. ^72^ However, reports of the China Health and Nutrition Survey showed that potassium consumption increased by 0.3 g/d over an 18-year period. However, the intake level is still lower than the recommendation. Women, low-income groups, and lower-education groups showed a lower intake of potassium. ^16^^,^^17^ Low potassium intake is usually due to low fruit and vegetable intake. Moreover, the recent studies showed potassium intake is associated with nutritional quality and food cost, so the cost of food often may inhibit its intake. ^14^^,^^73^ Low potassium consumption has also been observed in healthy Italian young people (aged 6–18 years), with a daily potassium intake of nearly 40 mmol, like in conformity with 1.5 g/day. ^74^ Recent international and national surveys have revealed that the majority of populations across the world consume less than the advocated levels of potassium. Collectively, these discoveries indicate the requirements for concerted attempts to promote higher potassium intake across the world.

### Sodium to potassium (Na/K) ratio

We extracted the Na/K ratio from 27 (63%) out of 43 studies belonging to the meta-analysis; the mean Na/K ratio was 3.38 (95% CI, 2.68–4.08) while the mean Na/K ratio in children was 3.61 (95% CI, 3.07–4.14). In the study by Du *et al.* (2014), ^17^ the mean Na/K ratio in 2009 was 2.8, more than half of subjects had a Na/K ratio above 2.0. It is important to note that the Na/K ratio was much over the recommended level. Sodium potassium ratio of 1 is considered beneficial for health. ^15^ Therefore, achieving effective ways to lower this ratio in the population by reducing dietary consumption of sodium and promoting dietary consumption of potassium is a key imperative since there are no reports of potassium toxicity due to dietary consumption. ^16^

Several studies have shown a correlation of the Na/K ratio with health outcomes. The dietary Na/K ratio was found to be a significant risk factor for mortality from stroke, cardiovascular disease, and all causes among the Japanese population. ^59^ Reduction in blood pressure showed a significant correlation with reduced a urinary Na/K ratio and improved urinary potassium. ^75^ Epidemiological research advises that the urinary Na/K ratio can be an advanced metric as compared to sodium and potassium levels alone for determining the association to BP and risk of cardiovascular disorder. ^58^ In some studies, the Na/K ratio was inversely related to SBP; however, those outcomes have been commonly weaker in comparison with the outcomes of potassium alone. ^76^ Farapti *et al*. (2017) ^11^ found that the Na/K ratio may be a beneficial marker for predicting BP in a specific population. In addition, to hypertension, the Na/K ratio was also indicated to be related to obesity. Another study by Cai *et al.* (2016) ^77^ also found an association between urinary Na/K ratio and obesity.

### Dietary intake of sodium and potassium as a community health problem

Several epidemiological studies and systematic reviews have found an association between low potassium consumption and high sodium consumption with several non-communicable diseases such as hypertension, ^4^ obesity, ^9^ cardiovascular diseases, ^78^^,^^79^ strokes, ^10^ chronic kidney disease, ^80^ and also all-cause mortality. ^10^ Our systematic review only pertained to subjects in a community setting and not hospitalized patients; several studies have analyzed the affiliation of these nutrients with blood pressure and/or obesity at the population level.

Some research has discovered no significant association with sodium and/or potassium intake together with BP, ^37^^–^^39^^,^^43^ however, a majority of studies and some systematic reviews and meta-analyses of observational research have found a significant relationship among children, adults, and the elderly. According to a systematic review by Newberry *et al*. (2018), ^81^ lowering sodium intake, increasing potassium intake, and the use of potassium-containing salt substitutes within the meals may significantly lower the BP, in particular among those with high blood pressure. Furthermore, a systematic review of studies published during 1995-2001 examined the influence of lowering sodium intake or potassium supplementation on BP. In all meta-analyses, the decrease in BP was found to be larger in hypertensive than normotensive subjects. ^75^^,^^82^^,^^83^ A systematic review of another dietary intervention reported that the Dietary Approaches to Stop Hypertension (DASH) diet (low sodium and high potassium diet) had the most important net impact on reducing BP. ^84^ Finally, an umbrella review showed that the DASH dietary pattern is related to lowered incidences of cardiovascular illnesses and improved BP. ^85^

The significant impact of sodium and potassium intake on BP has also been demonstrated in children and adolescents. ^85^^,^^86^ Excessive sodium consumption is a cause of increased BP in adults, youngsters, and teenagers. A systematic review of evidence from experimental and observational studies showed a positive relationship between sodium intake and BP in children and adolescents; consistent findings were obtained from experimental and observational studies. ^86^ On the other hand, another study showed the intake of higher potassium-rich foods in childhood may help prevent increased BP in adolescence. ^76^

Ma *et al.* (2015) ^87^ analyzed the UK National Diet and Nutrition Survey (2008/2009 to 2011/2012) data which result showed higher salt intake in overweight and obese subjects. In a study by Elfassy *et al.* (2018), ^88^ sodium intake was related to elevated body mass index (BMI), waist circumference (WC), and body fat content. According to the National Health and Nutrition Examination Survey (NHANES; 1999–2006), high sodium intake (>2300 mg/d) was related to a higher risk of obesity compared with intermediate sodium consumption (1500–2300 mg/d). ^89^

Some studies have revealed the affiliation between sodium or salt intake and obesity. However, not many researchers have reported the relation between potassium consumption and obesity. In s study by Cai *et al.* (2016), ^77^ high potassium consumption was not related to a lowered risk of obesity, although potassium consumption was related to metabolic syndrome. Furthermore, the urinary Na/K ratio was related to obesity. In a recent study by Tal *et al.* (2019), ^90^ subjects who achieved a decrease in BMI showed an average increase in potassium intake by 25%. Most studies that analyzed the correlation between these nutrients (sodium and potassium) and obesity were performed in children.

Importantly, several studies assessed the correlation of sodium and potassium consumption with both obesity and high blood pressure. Leyvraz *et al.* (2018) ^86^ observed that the correlation between sodium consumption and BP became more prominent in children who were overweight and low in potassium levels. In a study of children living in a Canadian rural community, being overweight or obese were strongly associated with elevated BP. ^91^ Delmis (2010) ^92^ found that overweight and obese children were at a 3–5 fold increased risk of hypertension. Concerning to the adult population, the NHANES (1999-2006) with 9162 healthy participants showed an independent correlation of high sodium intake with increased risk of both android and gynoid fat storage in the USA working age population. ^89^

## Conclusion

The present study highlights that the adult population in most parts of the world continues to consume excessively high levels of sodium and low levels of potassium. This phenomenon was reflected in the Na/K ratio exceeding the current recommended level. This dietary pattern is correlated with an elevated risk of hypertension and obesity. Our study underlines the excessive dietary intake of sodium and low intake of potassium as a global public health problem and more strategic intervention possibly at policy level is warranted.

## Data availability

### Underlying data

All data underlying the results are available as part of the article and no additional source data are required.

### Extended data

OSF: Extended data for: “Community-level dietary intake of sodium, potassium, and sodium-to-potassium ratio as a global public health problem: A systematic review and meta-analysis”, http://doi.org/10.17605/OSF.IO/976RS. ^93^

This project contains the following extended data:

• Supplementary Material.docx

## Reporting guidelines

OSF: Extended data for: “Community-level dietary intake of sodium, potassium, and sodium-to-potassium ratio as a global public health problem: A systematic review and meta-analysis”, http://doi.org/10.17605/OSF.IO/UBPT3. ^94^

• PRIMSA checklist
• PRISMA flow diagram

Data are available under the terms of the Creative Commons Zero “No rights reserved” data waiver (CC0 1.0 Public domain dedication).
