# Supplementary material for: Community-level dietary intake of sodium, potassium, and sodium-to-potassium ratio as a global public health problem: a systematic review and meta-analysis
Source: F1000Res. 2025 Apr 22;11:953. Originally published 2022 Aug 18. [Version 3] doi: 10.12688/f1000research.122560.3 (PMC11969137; doi:10.12688/f1000research.122560.3)
Supplement: Peer review history

# Reviewer report: Reviewer response for version 2

**DOI:** 10.5256/f1000research.170548.r372336

**Published:** 2025-04-03

**Authors:** Jianjun Mu

**Affiliations:** 1 First Affiliated Hospital of Xi'an Jiaotong University, Xi'an, China

**Review Comments**

 This study employs systematic review and meta-analysis methods to assess urinary sodium and potassium excretion and their ratio on a global scale. The research topic is of significant importance, the methodology is rigorous, and the conclusions provide valuable practical guidance. The imbalance in dietary sodium and potassium intake is a widespread issue. Numerous studies have demonstrated that interventions in dietary sodium and potassium intake can significantly improve health outcomes, highlighting the importance of managing sodium and potassium intake. However, despite considerable efforts and implementation of various policies and measures by many countries, significant disparities in sodium and potassium intake management persist, revealing global inequalities. The disease burden associated with sodium and potassium intake requires ongoing attention and resolution. This study is problem-oriented, and its results provide important evidence for improving the monitoring system and screening strategies for sodium and potassium management. The analysis of the sodium-potassium ratio and the subgroup analysis by continent are highlights of this study, deepening our understanding of the global health inequality burden.

**Minor remarks:**

 In the statistical analysis section, it is mentioned: "Subsequently, subgroup analysis was conducted to specify sodium and potassium excretion disaggregated by age-group (children and adults) and geographical location (grouped by continent)." The authors mention conducting a subgroup analysis by age, but the results seem to be missing from the main text. Please include this information in the manuscript.

 In the "Sodium to potassium (Na/K) ratio" section, the authors state: "In some studies, the Na/K ratio was inversely related to SBP; however, those outcomes have been commonly weaker in comparison with the outcomes of potassium alone". There is a small error here. According to the cited literature, the correct statement should be: "While the potassium to sodium ratio was also inversely associated with systolic blood pressure (P = .04), these effects were generally weaker compared with effects for potassium alone."

 Language expression: The overall language of the article is fluent, but there are still some minor errors and areas that could be more coherent. It is recommended to seek further polishing from a professional academic editor or native English speaker to improve the language quality of the article.

 Overall evaluation: This is a high-quality research paper with important academic value and practical significance. If the above issues can be addressed and improved, it will further enhance the quality and potential impact of the article. Agree to publish.

Are the rationale for, and objectives of, the Systematic Review clearly stated?

Yes

Is the statistical analysis and its interpretation appropriate?

Yes

If this is a Living Systematic Review, is the ‘living’ method appropriate and is the search schedule clearly defined and justified? (‘Living Systematic Review’ or a variation of this term should be included in the title.)

Yes

Are sufficient details of the methods and analysis provided to allow replication by others?

Yes

Are the conclusions drawn adequately supported by the results presented in the review?

Yes

Reviewer Expertise:

NA

I confirm that I have read this submission and believe that I have an appropriate level of expertise to confirm that it is of an acceptable scientific standard.

# Reviewer report: Reviewer response for version 1

**DOI:** 10.5256/f1000research.134564.r298775

**Published:** 2024-08-26

**Authors:** Ines Drenjančević

**Affiliations:** 1 University of Josip Juraj Strossmayer, Osijek, Croatia

This is systematic review and meta analysis of a number of studies related to dietary intake of sodium, potassium and calculated sodium-to-potassium ratio. The study is performed by review of publications in various indexing basis. The study is conducted appropriately.

**Minor remarks:**

- Why no studies from South America or Canada were included in analysis?
- If two studies that used spot-urine sample were excluded from analysis, would obtained data be different?
- Kindly provide in Discussion section data from WHO on Na, K intake and compare that to your data?
- In Discussion section, please provide or highlight  the arguments for Conclusion that "...This dietary pattern is correlated with an elevated risk of hypertension and obesity. "- e.g. provide a table or infformation from analyzed studies.

Are the rationale for, and objectives of, the Systematic Review clearly stated?

Yes

Is the statistical analysis and its interpretation appropriate?

Partly

If this is a Living Systematic Review, is the ‘living’ method appropriate and is the search schedule clearly defined and justified? (‘Living Systematic Review’ or a variation of this term should be included in the title.)

Not applicable

Are sufficient details of the methods and analysis provided to allow replication by others?

Yes

Are the conclusions drawn adequately supported by the results presented in the review?

Yes

Reviewer Expertise:

microcirculation, sodium/potassium dietary intake, physiology, immunology, vascular reactivity

I confirm that I have read this submission and believe that I have an appropriate level of expertise to confirm that it is of an acceptable scientific standard, however I have significant reservations, as outlined above.

# Response

**Published:** 2025-04-15

**Authors:** Farapti Farapti

**Affiliations:** Faculty of Public Health, Indonesia

**Reviewer :**

 Why no studies from South America or Canada were included in analysis?

**Author response :**

 The systematic review does not provide a specific explanation for the absence of studies from South America or Canada in the analysis. However, it is common in systematic reviews that the inclusion of studies is influenced by the availability of relevant research published within the  specified time frame and the search criteria used. The authors may not have found studies that met eligibility criteria from these regions during literature search, which focused on observational studies published between January 2014 and December 2021.

**Reviewer :**

 If two studies that used spot-urine sample were excluded from analysis, would obtained data be different?

**Author response :**

 The systematic review does not provide a specific explanation for the absence of studies from South America or Canada in the analysis. However, it is common in systematic reviews that the inclusion of studies is influenced by the availability of relevant research published within the  specified time frame and the search criteria used. The authors may not have found studies that met eligibility criteria from these regions during literature search, which focused on observational studies published between January 2014 and December 2021.

 Additionally, the systematic review aimed to include studies that reported 24-hour urinary excretion of sodium and potassium, and it is possible that such studies were limited or not published in the databases searched for these specific regions. Further details would likely be found in the discussion section of the paper, where the authors might address limitations related to geographic representation findings.

**Reviewer :**

 Kindly provide in Discussion section data from WHO on Na, K intake and compare that to your data?

**Author response :**

 Thank you for the suggestion.

 We added the discussion about WHO recommendation of sodium intake and compared to our data

**Reviewer :**

 In Discussion section, please provide or highlight  the arguments for Conclusion that "...This dietary pattern is correlated with an elevated risk of hypertension and obesity. "- e.g. provide a table or infformation from analyzed studies.

**Author response :**

 Thank you for the suggestion

 We noted it as the limitation of this study and added the information in the last paragraph of discussion section.

 In this study, we attempt to systematically investigate the dietary consumption of sodium and potassium among adults at the community level across the world.The objective was to quantify the dietary intake of sodium and potassium as a public health problem at the community level.

 In the discussion section of dietary intake of sodium and potassium as a community health problem, the discussion would likely emphasize that the high sodium and low potassium intake patterns observed in their study correlate with increased risks of hypertension and obesity as public health problem in community setting. However, we did not calculate details of  quantity by meta-analysis table or figure, we only explain clearly some studies related it.

# Reviewer report: Reviewer response for version 1

**DOI:** 10.5256/f1000research.134564.r243799

**Published:** 2024-03-05

**Authors:** Nan Xin Wang

**Affiliations:** 1 Department of Human Nutrition, University of Otago, Dunedin, Otago, New Zealand

This systematic review investigated sodium and potassium intake in healthy adults using observational studies that reported 24-hour urinary excretion of sodium and potassium.

 Introduction:

 Para 1: There is a strong emphasis on Asian diets transiting to "western" diet. However, the article focus on worldwide. Typically, Asian countries have higher sodium intake than European, American and Australia/NZ. So, it seems incorrect to comment that the adoption of Westernised diet contributes to increased sodium intake. It is the adoption of convenience and ultra-processed foods that contributes to higher sodium intake. So, it is unclear why the authors have singled Asian diets. 

 Lifestyle: would be cover much wider aspect of life than just dietary pattern, please change the wording. (Similarly, please remove from abstract.)

 Para 3: WHO recommendations are not strategic interventions. These are guidelines for interventions which countries can adopt. Perhaps, you could describe nation-wide interventions and its effects on intake.

 Para 4: More details is needed on the systematic review conducted in China, how does this relate to the current paper? 

 Methods: PRISMA is reportedly followed, please include the PRISMA table as supplementary table. 

 Results: I am unable to access the supplementary table for study characteristics. 

 Para 2: please follow spelling intervention, for numbers ten and below, please spell in full. (Please follow this convention throughout the article)

*"Likewise,* *the studies by Du et al. (2014)* *17* *and Morrisey et al. (2020)* *34* *used the spot urine method in combination with a dietary* *survey." *This sentence is not a result of the study. Please move it to discussion. It confuses the reader.

 Para 4: summaries, should be summarises

 remove presents.

 please use urinary excretion throughout

 Discussion:

 Para 1: Please elaborate on reliable and convenient methods.

 Para 2: Sentence 1, replace was with were.

 Please elaborate what kinds of research were included in Tan et al.

 Para 3: Please state if the numbers in brackets are 95% CI, min/max, 25th/95th percentile. Sentence 3, replace which with who.

 Please state the recommendations of IOM.  Please be consistent in using mg or mmol. Alternatively, provide both units of measurement. 

 Sentence 9, replace seems with seem.

 Please expand the two final sentence, what do you mean by " *well greater* than minimal physiological requirements"? More concrete examples are required. 

 Para 4: suggest to reword, :" *However, sodium is a naturally occurring nutrient into a vast range* over foods"

 Although sodium is naturally occurring in foods, most sodium is added during processing. Hence, this in itself should not be much of an issue with sodium intake.

 Please type in full *"they*’re". 

 Para 5: Please provide estimate for Potassium intake in Benin and describe how potassium intake was assessed.

 Para 11 and para 12: Suggest to reorganise.

 Conclusion: " *This dietary pattern is correlated with an elevated risk of hypertension and obesity." *This is not part of the study's results and therefore, cannot be concluded as such.

Are the rationale for, and objectives of, the Systematic Review clearly stated?

Partly

Is the statistical analysis and its interpretation appropriate?

I cannot comment. A qualified statistician is required.

Are sufficient details of the methods and analysis provided to allow replication by others?

Yes

Are the conclusions drawn adequately supported by the results presented in the review?

Partly

Reviewer Expertise:

Sodium, policy, iodine

I confirm that I have read this submission and believe that I have an appropriate level of expertise to confirm that it is of an acceptable scientific standard, however I have significant reservations, as outlined above.

# Response

**Published:** 2024-08-17

**Authors:** Farapti Farapti

**Affiliations:** Faculty of Public Health, Indonesia

**ABSTRACT AND INTRODUCTION**

**Reviewer:**

 Lifestyle: would be cover much wider aspect of life than just dietary pattern, please change the wording. (Similarly, please remove from abstract.)

**Author Response:**

 You're right; lifestyle encompasses a much broader spectrum of life factors beyond just dietary patterns. This includes aspects like physical activity, stress levels, sleep habits, and more. Thank you for emphasizing the need to consider these broader aspects when discussing health impacts.

 Our suggestion: changing "Lifestyle" to "eating habit"

**Reviewer:**

 Para 1: There is a strong emphasis on Asian diets transiting to "western" diet. However, the article focus on worldwide. Typically, Asian countries have higher sodium intake than European, American and Australia/NZ. So, it seems incorrect to comment that the adoption of Westernised diet contributes to increased sodium intake. It is the adoption of convenience and ultra-processed foods that contributes to higher sodium intake. So, it is unclear why the authors have singled Asian diets.

 Lifestyle: would be cover much wider aspect of life than just dietary pattern, please change the wording. (Similarly, please remove from abstract.)

**Author Response:**

 Thank you for highlighting valid point about the transition to "western" diets not being solely responsible for increased sodium intake. The emphasis should indeed be on the shift towards convenience and ultra-processed foods rather than singling out Asian diets. It's important to consider the broader global dietary trends and their impact on health. Thank you for highlighting this perspective.

 You're right; lifestyle encompasses a much broader spectrum of life factors beyond just dietary patterns. This includes aspects like physical activity, stress levels, sleep habits, and more. Thank you for emphasizing the need to consider these broader aspects when discussing health impacts.

 Our suggestion: changing "Lifestyle" to "eating habit"

**Reviewer:**

 Para 3: WHO recommendations are not strategic interventions. These are guidelines for interventions which countries can adopt. Perhaps, you could describe nation-wide interventions and its effects on intake.

**Author Response:**

 Thanks for your advice. We have describe in several countries regarding nation-wide interventions about sodium potassium

**Reviewer:**

 Para 4: More details is needed on the systematic review conducted in China, how does this relate to the current paper? 

**Author Response:**

 The research in China focused on the domestic population, so it became a reference for us to systematically research dietary consumption of Na/K in various countries from all over the world. So it is hoped that the results of this research can also become knowledge that can be accessed more widely.

**METHODS**

**Reviewer:**

 Methods: PRISMA is reportedly followed, please include the PRISMA table as supplementary table.

**Feedback:**

 Thank you, we have added the PRISMA table in the supplementary file

**RESULTS**

**Reviewer:**

 Results: I am unable to access the supplementary table for study characteristics. 

**Author Response:**

 Thank you, we have added the study characteristics table in the supplementary file

**Reviewer:**

 Para 2: please follow spelling intervention, for numbers ten and below, please spell in full. (Please follow this convention throughout the article)

*"Likewise,* *the studies by Du et al. (2014)17 and Morrisey et al. (2020)34 used the spot urine method in combination with a dietary* *survey." *This sentence is not a result of the study. Please move it to discussion. It confuses the reader.

**Author Response:**

 Thank you, we have revised that points

**Reviewer:**

 Para 4: summaries, should be summarises remove presents. please use urinary excretion throughout

**Author Response:**

 Thank you, we have removed presents and only use urinary excretion throughout

**DISCUSSION:**

**Reviewer:**

 Para 1: Please elaborate on reliable and convenient methods.

**Author Response:**

 Thank you for the suggestion. We rearranged some sentences to elaborate clearly on reliable and convenient methods.

**Reviewer:**

 Para 2: Sentence 1, replace was with were.

 Please elaborate what kinds of research were included in Tan et al.

**Author Response:**

 The eligible studies by Tan et al were they were conducted only in China (Chinese population), no restriction on study year and subjects’age, applied 24-hor urine collection to assess Na and K intake

**Reviewer:**

 Para 3: Please state if the numbers in brackets are 95% CI, min/max, 25th/95th percentile. Sentence 3, replace which with who.

**Author Response:**

 We stated the number in brackets are 95%CI (min-max). We revised which with who.

**Reviewer:**

 Please state the recommendations of IOM.  

**Author Response:**

 We stated the recommendation of IOM about sodium intake

**Reviewer:**

 Please be consistent in using mg or mmol. Alternatively, provide both units of measurement.

**Author Response:**

 Thank you for the suggestion

 We changed the mg with mmol. We will consistent in using mmol for measurement unit of sodium intake

**Reviewer:**

 Sentence 9, replace seems with seem.

**Author Response:**

 We revised seems with seem

**Reviewer:**

 Please expand the two final sentence, what do you mean by " *well greater* than minimal physiological requirements"? More concrete examples are required.

**Author Response:**

 Thank you for the suggestion.

 We deleted the two final sentence, and arranged the new sentences to explain clearly and give concrete example about basic nutritional requirements for sodium intake

**Reviewer:**

 Para 4: suggest to reword, :" *However, sodium is a naturally occurring nutrient into a vast range* over foods"

 Although sodium is naturally occurring in foods, most sodium is added during processing. Hence, this in itself should not be much of an issue with sodium intake.

 Please type in full *"they*’re". 

**Author Response:**

 Thank you for the correction.

 We reworded as your suggestion

 We corrected and changed they’re with they are

**Reviewer:**

 Para 5: Please provide estimate for Potassium intake in Benin and describe how potassium intake was assessed.

**Author Response:**

 We would like to clarify that we would like to point out that the study in Benin showed a high prevalence of hypertension

**Reviewer:**

 Para 11 and para 12: Suggest to reorganise.

**Author Response:**

 Thank you for the suggestion

 We arranged and organized two paragraphs become one paragraph discussing about sodium and potassium intake and the correlation with obesity

**CONCLUSION**

**Reviewer:**

 Conclusion: " *This dietary pattern is correlated with an elevated risk of hypertension and obesity." *This is not part of the study's results and therefore, cannot be concluded as such.

**Author Response:**

 Thank you for the correction. We deleted the sentence
